# Factors affecting the preference of anesthesia residents regarding subspecialty training

**DOI:** 10.1186/s12909-019-1782-9

**Published:** 2019-09-06

**Authors:** Murat Izgi, Betul Basaran, Aysun Ankay Yilbas, Sennur Uzun, Almıla Gulsun Pamuk, Meral Kanbak

**Affiliations:** 1 Faculty of Medicine, Department of Anesthesiology and Reanimation, Hacettepe University, Altindag, Ankara, Turkey; 2 Konya Training and Research Hospital, Clinics of Anesthesiology and Reanimation, University of Health Sciences, Konya, Turkey

**Keywords:** Anesthesia residents, Preferences, Subspecialty training

## Abstract

**Background:**

There is scant information about the factors that influence the career decisions of anesthesia residents in Turkey. The aim of this study was to determine the preferences of anesthesia residents in Turkey regarding future career and subspecialty training plans and practice location, and to determine the factors that influence those preferences.

**Methods:**

A 21-item e-questionnaire was administered to anesthesia residents who were registered with at least one of the two societies of anesthesiologists in Turkey. Data were collected on demographics and preferences regarding subspecialty training and future practice location.

**Results:**

The response rate of the survey was 41.04%. The percentages of participants who intended to pursue a fellowship in intensive care and algology were 12.1 and 23.1%, respectively; 21.7% of participants did not intend to pursue any fellowship training and the decision of 43.1% of the participants was uncertain. The most popular reasons for pursuing a fellowship were to perform compulsory service in a better place (47.2%) and improve earning potential (43.1%). Forty-two percent of participants did not intend to pursue any fellowship training because of their attention to general anesthesiology practice and 15.2% because of the additional compulsory service obligation following the training.

**Conclusions:**

This study showed that the desirability of sub-specialization among anesthesia residents in Turkey could be accepted as low. This result seems to be associated with the additional compulsory service obligation.

**Electronic supplementary material:**

The online version of this article (10.1186/s12909-019-1782-9) contains supplementary material, which is available to authorized users.

## Background

Anesthesia residency in Turkey lasts for 5 years, following 6 years in medical school. At the end of this education period and following compulsory services for both levels of education, anesthetists have two alternatives for their occupational future; pursuing a subspecialty (algology or intensive care) or working as an anesthesiologist. If they choose to pursue a fellowship, they have to pass the fellowship examination, which is performed once a year after finishing the residency period. Also, there is a third compulsory service period following the subspecialty training. Compulsory service is used in more than 70 countries to ensure sufficient and balanced distribution of physicians and to obtain positive healthcare outcomes. The first application of compulsory service in the literature was in Russia in 1920 [1]. The currently applied compulsory service program in Turkey started in 2005, one that is somewhat unique because physicians cannot gain a license to practice before they complete their compulsory service, which includes working in underdeveloped regions for a period of 300 to 600 days. Physicians must perform compulsory service after every training period; first after medical school, second if they pursue a residency to be a specialist, and third after the fellowship training period if they want to be a subspecialist. Therefore, compulsory service constitutes an extraordinary factor that affects the choice of whether to undertake subspecialty education among anesthesiologists in Turkey.

Factors influencing decision-making vary by country and division. The most influential factor in pursuing a subspecialty for Canadian anesthesia residents was personal interest [2]. A similar study from Turkey reported that the most influential factors in pursuing a subspecialty for physical and rehabilitation medicine residents in Turkey were prestige and the possibility of performing compulsory service in a better location [3]. Determining the underlying reasons for residents’ future career plans seems precious for the planners of the future of the profession.

There is scant information about factors that influence the career decisions of residents in Turkey. Up to now, there have been no studies regarding anesthesia subspecialty training in our country so we aimed to determine the preferences of anesthesia residents in Turkey for their future career, subspecialty plans, practice location, and to determine factors that influenced those preferences. We hypothesized that compulsory service could also have an impact on the preferences of anesthesia residents in Turkey.

## Methods

A 21-item e-questionnaire (see Additional file 1) generated using Google Forms (Mountain View, California, USA) was applied to anesthesia residents who were registered with at least one of the two societies of anesthesiologists in Turkey. The study was conducted in accordance with the principles of the Declaration of Helsinki after obtaining ethical approval of the Non-interventional Clinical Research Ethics Committee of Hacettepe University. According to data from “The historical development of anesthesiology and reanimation science in Turkey, volume II” report, there were approximately 1208 anesthesia residents in Turkey [4]. Six hundred ninety-two of those residents were registered in Turkish Society of Anesthesiology and Reanimation (TARD) and the Anesthesiology and Reanimation Specialists Society (ARUD), the two main anesthesiology and reanimation societies in Turkey. We sent an e-mail and link for the survey to all of registered residents via TARD and ARUD. The survey included 4 sections: 1) demographic information, 2) presence of subspecialty divisions, 3) decision about pursuing fellowship training, type of fellowship, and influential factors about this decision, and 4) factors influencing the location choice for fellowship training and for practice location after fellowship training.

The questionnaire was based on surveys conducted among anesthesiology residents in Canada and residents of physical and rehabilitation medicine in Turkey [2, 3] after consultation with an anesthesia residency program director and fellowship program director. Thirty anesthesia residents then piloted the questionnaire to ensure clarity and ease of completion. We improved our survey and revised the unclear questions. Accordingly, using the results of this pilot study, we created the questionnaire with 21 questions, which is attached in the appendix.

### Statistical analysis

Whether the distributions of continuous variables were normal was determined using the Kolmogorov-Smirnov test. The Levene test was used for the evaluation of homogeneity of variances. Descriptive statistics are shown as mean ± standard deviation (SD) or number of cases and (%), where applicable.

The mean differences in ages between the groups were compared using Student’s t-test, and one-way analysis of variance (ANOVA) was applied for comparisons among more than two independent groups. The differences in durations of post-graduate education were evaluated using the Mann- Whitney U or Kruskal = Wallis test, where appropriate. When the *p*-value from the Kruskal-Wallis test was statistically significant, Conover’s multiple comparison tests was used to determine which group differed from the others. Categorical data were analyzed by Pearson’s Chi-square test.

Whether the associations between the responders’ characteristics and decisiveness for pursuing fellowship training were statistically significant was examined by univariate logistic regression analyses. Determining the best predictor(s) that affect the decision for pursuing fellowship training was evaluated using multiple logistic regression analysis. The factors that were thought to be the most effective in distinguishing among the responders (i.e. negatively, positively, and undecided) were determined using multinomial logistic regression analyses. Any variable whose univariable test had a *p*-value < 0.25 was accepted as a candidate for the multivariable model along with all variables of known clinical importance. Odds ratios and 95% confidence intervals for each independent variable were also calculated.

Data analysis was performed using IBM SPSS Statistics version 17.0 software (IBM Corporation, Armonk, NY, USA). A *p* value less than 0.05 was considered statistically significant.

## Results

### Demographics

There were 1208 anesthesia residents in the university hospitals’ and training hospitals’ anesthesiology and reanimation clinics/departments of the country when we performed the study. Of the 692 residents registered (57.3% of all residents) with at least one of the societies of Anesthesiology and Reanimation in Turkey, 284 residents responded to the survey (41.04% response rate, 23.5% of all anesthesia residents in Turkey). Three of the respondents did not answer most of the questions and were excluded from further analysis, yielding 281 residents (161 females, 120 males) for analysis. The mean age of respondents was 29.8 ± 3.9 years. Of the responses provided by the residents of all five postgraduate years, 25.4% were second and 20.4% were fourth-year postgraduate residents. The descriptive statistics of the participants’ demographic characteristics are shown in Table 1.

**Table 1:** Demographic information of the respondents

Variables	Statistics
Sex, n (%)	
Female	161 (57.3)
Male	120 (42.7)
Age (years), mean ± SD	29.8 ± 3.9
Marital Status, n (%)	
Single	135 (48.0)
Married	146 (52.0)
Type of Hospital of Residency, n (%)	
University Hospital	155 (55.4)
Education and Research Hospital (Ministry of Health)	125 (44.6)
Post Graduate Year, n (%)	
1st year	55 (19.7)
2nd year	71 (25.4)
3rd year	53 (19.0)
4th year	57 (20.4)
5th year	43 (15.4)
Any participation of a study during residency except thesis, n (%)	
No	160 (57.1)
Yes	120 (42.9)
Any published article during residency, n (%)	
No	236 (84.0)
Yes	45 (16.0)
The existence of Algology unit, n (%)	
No	82 (29.2)
Yes	199 (70.8)
The effects of the existence or absence of the subspecialty unit, n (%)	
No	97 (34.5)
Yes	184 (65.5)

### Subspecialty decision

**Fig. 1:**
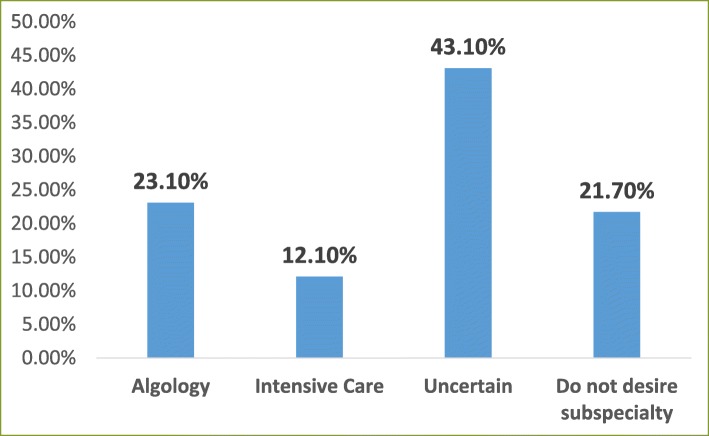
Subspecialty preference

**Fig. 2:**
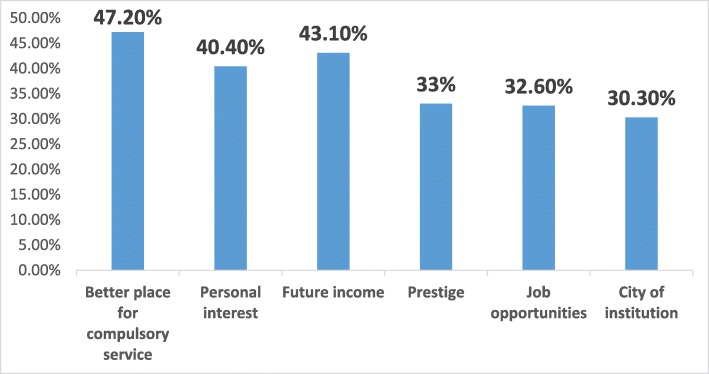
Influencing factors of pursuing a fellowship

**Fig. 3:**
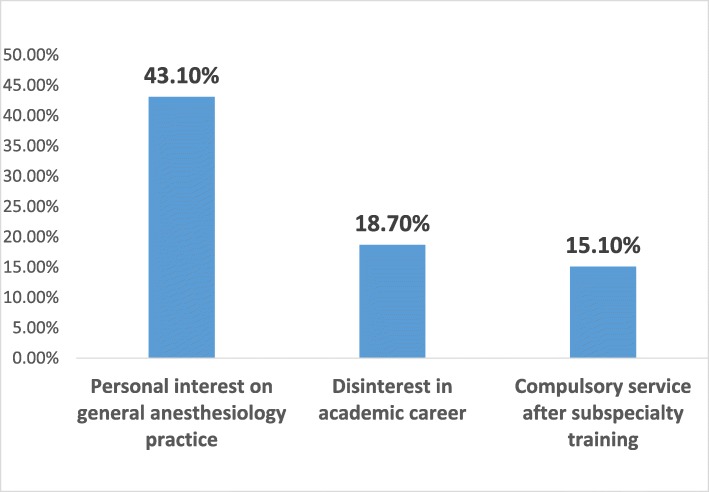
Influencing factors of not pursuing a fellowship

**Fig. 4:**
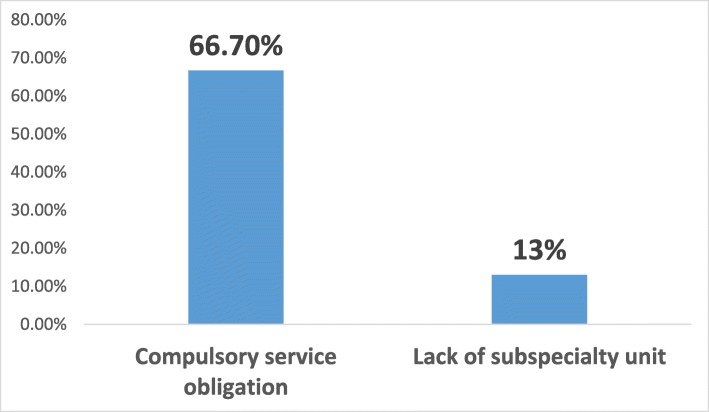
Causes of uncertainty about subspecialty training

**Fig. 5:**
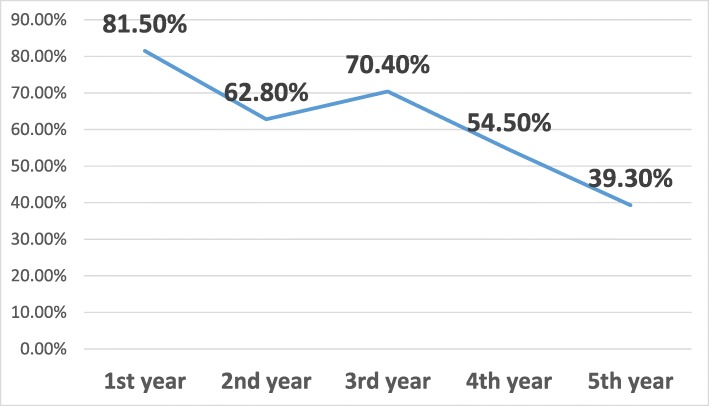
Relationship between post-graduate year and desire of subspecialty training

The subspecialties of anesthesiology are algology and intensive care in Turkey. We asked the residents whether they wanted to pursue a fellowship program and 21.7% responded negatively, 35.2% did plan to pursue a fellowship (23.1% algology and 12.1% intensive care), and 43.1% were undecided (Fig. 1). Of those who wanted to pursue a fellowship program, nearly two-thirds of the respondents wanted algology fellowship programs and nearly one-third of the respondents wanted intensive care fellowship programs. Intensive care units were present in 100% of the respondents’ hospitals, whereas algology units were present in 70.5%. Nearly two-thirds of the respondents declared that the presence or existence of the subspecialty unit in their hospital affected their decision. Among those who decided to pursue a fellowship program, the most common factors affecting this decision were to work in a better place during compulsory service (47.2%), improve earning potential (43.1%), and personal interest (40.4%) (Fig. 2). Personal interest in general anesthesia (43.1%), disinterest in an academic career (18.7%), and compulsory service following the fellowship training (15.1%) were the most popular reasons of the residents who did not plan to pursue a fellowship program (Fig. 3). The most affective factor for the residents who were undecided about pursuing a fellowship program was performing compulsory service (66.7%) and the lack of the subspecialty unit at the hospital (13%) at which that they worked during the residency period (Fig. 4). The postgraduate year of the respondents was negatively associated with the desire to pursue fellowship training (Fig. 5).

The comparisons of the demographic characteristics of the participants according to their wish to pursue a fellowship are shown in Table 2. The percentage of males was statistically higher and the percentage of females was statistically lower in the group who wanted to pursue subspecialty training compared with the residents who did not want (*p* = 0.026) and were undecided about whether to pursue subspecialty training (*p* = 0.042). The distribution of men and women was statistically similar among the groups that did not want to pursue subspecialty training and that were undecided about whether to pursue subspecialty training (*p* = 0.561). There was a statistically significant difference between the groups in terms of the distribution of duration of residency (*p* = 0.004). The group that did not want to pursue subspecialty training had been working for a longer time than other groups (*p* = 0.002 and *p* = 0.004). In the group that wanted to pursue subspecialty training, the percentage of those who had published articles was higher than those who were undecided (*p* = 0.013). Significantly more residents in the groups that wanted to pursue subspecialty training and that were undecided declared that the existence or absence of the subspecialty unit affected their decision (*p* < 0.001).

**Table 2:** Demographic information regarding for respondent groups

Variables	Residents who do not want to pursue fellowship	Residents who want to pursue fellowship	Undecided about pursuing a fellowship	*p*-value
Sex, n (%)				**0.042** ^†^
Female	40 (65.6)^a^	47 (47.5)^a,b^	74 (61.2)^b^	
Male	21 (34.4)^a^	52 (52.5)^a,b^	47 (38.8)^b^	
Age (years), mean ± SD	30.0 ± 3.3	30.0 ± 4.5	29.5 ± 3.7	0.561^‡^
Marital Status, n (%)				0.497^†^
Single	27 (44.3)	45 (45.5)	63 (52.1)	
Married	34 (55.7)	54 (54.5)	58 (47.9)	
Type of Hospital of Residency, n (%)				0.232^†^
University Hospital	32 (52.5)	61 (62.2)	62 (51.2)	
Education and Research Hospital	29 (47.5)	37 (37.8)	59 (48.8)	
Post Graduate Year, n (%)				**0.004** ^¶^
1st year	5 (8.2)	22 (22.7)	28 (23.1)	
2nd year	16 (26.2)	27 (27.8)^a^	28 (23.1)	
3rd year	8 (13.1)	19 (19.6)	26 (21.5)^c^	
4th year	15 (24.6)^a,c^	18 (18.6)	24 (19.8)	
5th year	17 (27.9)	11 (11.3)	15 (12.4)	
Any participation of a study during residency except thesis, n (%)				0.344^†^
No	30 (49.2)	60 (60.6)	70 (58.3)	
Yes	31 (50.8)	39 (39.4)	50 (41.7)	
Any published article during residency, n (%)				**0.041** ^†^
No	52 (85.2)	76 (76.8)^b^	108 (89.3)^b^	
Yes	9 (14.8)	23 (23.2)^b^	13 (10.7)^b^	
The existence of Algology unit, n (%)				0.782^†^
No	20 (32.8)	28 (28.3)	34 (28.1)	
Yes	41 (67.2)	71 (71.7)	87 (71.9)	
The effects of the existence or absence of the subspecialty unit, n (%)				**< 0.001** ^†^
No	36 (59.0)^a,c^	31 (31.3)^a^	30 (24.8)^c^	
Yes	25 (41.0)^a,c^	68 (68.7)^a^	91 (75.2)^c^	

^†^Pearson’s Chi-square test; ^‡^One-Way ANOVA; ^¶^Kruskal-Wallis test; ^a^The differences between negatively and positively responded groups were found as statistically significant (*p* < 0.05); ^b^The difference between positively responded and undecided groups were found as statistically significant (*p* < 0.05); ^c^The difference between negatively responded and undecided groups were found as statistically significant (*p* < 0.01)

Multinomial logistic regression analysis was performed to determine the most important factors in differentiating the groups according to the residents’ wish to pursue subspecialty training (Table 3). As a result of the univariate statistical analysis, the variables identified as *p* < 0.25 were included in the multinomial logistic regression model as predictors. The duration of the residency, the effect of the presence or absence of subspecialty units on the decision of pursuing subspecialty training, sex, and any published articles during the residency period were the most decisive factors, respectively. As the duration of the residency period extended, the desire to pursue subspecialty training decreased, independent of other factors (OR = 0.604, 95% CI: 0.459–0.795, *p* < 0.001). The existence or absence of subspecialty units in the institution where the residents’ worked was seen to have an effect on their decision (OR = 3.599, 95% CI: 1.761–7.355, *p* < 0.001). Independent of other factors, the desire to pursue subspecialty training in males was 2.550 times higher than in women (95% CI: 1.233–5.271, *p* = 0.012). Publishing an article during the residency period was found related to a 3.365 times greater desire to pursue subspecialty training, regardless of other factors (95% CI: 1.274–8.884, *p* = 0.014). The effect of the presence or absence of subspecialty units in the institution and the duration of the residency, respectively, were the most decisive factors that differentiated the participants who did not want to pursue subspecialty training and who were undecided. The effect of the presence or absence of subspecialty units in the institution in which the participants worked was found related to indecision about whether to pursue subspecialty training, independent of other factors (OR = 4.499, 95% CI: 2.284–8.864, *p* < 0.001). However, the likelihood of uncertainty decreased as the duration of the residency extended (OR = 0.713, 95% CI: 0.552–0.921, *p* = 0.009). Publishing an article during residency was found related to the decreased probability of indecision about subspecialty training (OR = 0.339, 95% CI: 0.150–0.767, *p* = 0.009).

**Table 3:** The results of multinomial logistic regression analyses in order to determine the best predictors which effect on discrimination of respondent groups

	Negatively responded vs positively responded		Negatively responded vs undecided		Positively responded vs undecided	
	OR (95% CI)	*p*-value	OR (95% CI)	*p*-value	OR (95% CI)	*p*-value
Male factor	2.550 (1.233–5.271)	**0.012**	1.495 (0.742–3.012)	0.261	0.586 (0.332–1.034)	0.065
University Hospital	1.224 (0.600–2.495)	0.578	0.835 (0.425–1.639)	0.600	0.682 (0.386–1.205)	0.188
Post Graduate Year	0.604 (0.459–0.795)	**< 0.001**	0.713 (0.552–0.921)	**0.009**	1.181 (0.945–1.477)	0.145
Any published article during residency	3.365 (1.274–8.884)	**0.014**	1.141 (0.422–3.088)	0.795	0.339 (0.150–0.767)	**0.009**
The existence or absence of the subspecialty unit	3.599 (1.761–7.355)	**< 0.001**	4.499 (2.284–8.864)	**< 0.001**	1.250 (0.673–2.325)	0.480

*OR* odds ratio, *CI* confidence interval

We also divided the participants into two groups according to whether they were undecided or decided about subspecialty training (whether positively or negatively). Having a published article during residency and the thought of the effect of the existence or absence of a subspecialty unit were the only two factors that were significantly different in the two groups (Tables 4 and 5).

**Table 4:** Demographic information regarding the decided and undecided respondent groups

Variables	Undecided about pursuing fellowship	Decided about pursuing fellowship	*p*-value
Sex, n (%)			0.255^†^
Female	74 (61.2)	87 (54.4)	
Male	47 (38.8)	73 (45.6)	
Age (years), mean ± SD	29.5 ± 3.7	30.0 ± 4.1	0.282^‡^
Marital Status, n (%)			0.240^†^
Single	63 (52.1)	72 (45.0)	
Married	58 (47.9)	88 (55.0)	
Type of Hospital of Residency, n (%)			0.227^†^
University Hospital	62 (51.2)	93 (58.5)	
Education and Research Hospital	59 (48.8)	66 (41.5)	
Post Graduate Year, n (%)			0.240^¶^
1st year	28 (23.1)	27 (17.1)	
2nd year	28 (23.1)	43 (27.2)	
3rd year	26 (21.5)	27 (17.1)	
4th year	24 (19.8)	33 (20.9)	
5th year	15 (12.4)	28 (17.7)	
Any participation of a study during residency except thesis, n (%)			0.727^†^
No	70 (58.3)	90 (56.3)	
Yes	50 (41.7)	70 (43.8)	
Any published article during residency, n (%)			**0.036** ^†^
No	108 (89.3)	128 (80.0)	
Yes	13 (10.7)	32 (20.0)	
The existence of Algology unit, n (%)			0.729^†^
No	34 (28.1)	48 (30.0)	
Yes	87 (71.9)	112 (70.0)	
The effects of the existence or absence of the subspecialty unit, n (%)			**0.003** ^†^
No	30 (24.8)	67 (41.9)	
Yes	91 (75.2)	93 (58.1)	

^†^Pearson’s Chi-square test, ^‡^Student’s t-test, ^¶^Mann-Whitney U test

**Table 5:** The results of univariate and multiple logistic regression analyses in order to determine the best predictors that affect the discrimination of decided and undecided responders

	Univariate analyses		Multivariate analysis	
	OR (95% CI)	*p*-value	OR (95% CI)	*p*-value
Male factor	1.321 (0.817–2.135)	0.256	–	–
Age	1.035 (0.972–1.103)	0.284	–	–
Married	1.328 (0.827–2.132)	0.241	1.237 (0.750–2.041)	0.405
University Hospital	1.341 (0.833–2.158)	0.227	1.430 (0.875–2.338)	0.154
Post Graduate Year	1.113 (0.934–1.327)	0.230	1.019 (0.839–1.236)	0.851
Any participation of a study during residency except thesis	1.089 (0.675–1.758)	0.727	–	–
Any published article during residency	2.077 (1.038–4.156)	**0.039**	1.832 (0.872–3.852)	0.110
The existence of Algology unit	0.912 (0.542–1.535)	0.729	–	–
The effects of the existence or absence of the subspecialty unit	0.458 (0.272–0.769)	**0.003**	0.459 (0.271–0.779)	**0.004**

*OR* odds ratio, *CI* confidence interval

### Location and hospital preference

When asked about the influencing factors on the location choice for subspecialty training after residency, the most popular reason was the city in which the hospital was located (68.2%) and the other most selected reasons were the reputation of the hospital (52.7%), the recommendation of the professors (36.4%), and family or personal factors (33.1%).

Regarding the preferences of the respondents about the hospital that they would like to practice in as a specialist after compulsory service, 53.6% planned to practice in an academic hospital, 25.7% planned to work in public hospitals, and 20.7% preferred to work in private hospitals. For the choice of the respondents about the type of hospital that they would want to work in as a sub-specialist after compulsory service, 65.7% planned to practice in an academic hospital, 8% wanted to practice in a public hospital, and 26.3% preferred private hospitals.

**Fig. 6:**
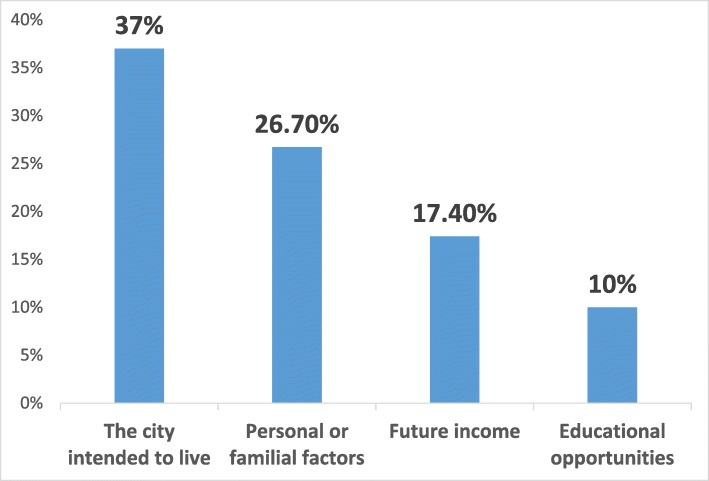
Influencing factors on the place to work as a specialist

The top four common factors that influence a resident’s preference for their desired future practice location (academic or community) were the city that they want to live in (37%), family or personal reasons (26.7%), economic income (17.4%) and educational opportunities (10%) (Fig. 6).

## Discussion

This study investigated the preferences and influential factors of anesthesia residents training in Turkey, a country that has an obligatory compulsory service programme, while making their future career plans. To our knowledge, no previous survey has been conducted in Turkey to explore which factors affected the preference of anesthesia residents regarding subspecialty training and their occupational career plans. With responses from many different institutions and a total 41.04% response rate with all levels of training, this study provides a snapshot of preferences and the affecting factors of anesthesia residents of Turkey.

The most influential factors on pursuing subspecialty were personal interest, enhancing employability, and an interest in an academic career for Canadian anesthesiology residents [2]. The only study performed in Turkey showed that the most influential factors of physical and rehabilitation medicine residents’ on planning to receive fellowship training were prestige (54.7%), being interested in an academic career (50%), and the possibility of performing compulsory service in a better location (34.4%) [3]. In our study, we found that the most influential factors on pursuing subspecialty were the possibility of performing compulsory service in a better location (47.2%), to improve earning potential (43.1%), and personal interest (40.4%). The Canadian anesthesiologists’ top reason for pursuing a fellowship and research was personal interest, whereas it was placed third for Turkish anesthesia residents [2]. This result shows that the existence of obligatory compulsory service in Turkey is a very important factor on the preferences of subspecialty for anesthesia residents.

The top reason of the residents for not desiring a subspecialty was personal interest in general anesthesiology (43.1%), whereas it was lack of interest for the Canadian colleagues [2]. If there were more and different subspecialties in our country, such as in Canada (e.g. regional anesthesia, pediatric anesthesia, obstetric anesthesia, cardiac anesthesia, neuroanesthesia), specialists could choose what they saw as closer to themselves. Thus, the percentage of those who might prefer to pursue subspecialty training would be increased. The next most important factors were disinterest in an academic career and subsequent compulsory service obligation, respectively. Also, the most important reason for uncertainty was the compulsory service obligation (66.7%), similar to physical and rehabilitation medicine residents in Turkey [3]. The reason for uncertainty about pursuing fellowship training of Canadian anesthesia residents was a lack of interest [2].

The main problem with the compulsory service program being applied in Turkey is the repetition following any level of education. Most of specialists are aged over 35 years, married, and have children. For every education and subsequent compulsory service periods, specialists need to re-establish all their future plans, which is why it is harder for physicians in Turkey to decide about pursuing fellowship programs following specialization. According to the results of this study, 35.2% of anesthesia residents plan to pursue fellowship training. This result is unexpectedly low when compared with the residents of anesthesia of Canada (70%) [2]. On the other hand, our results are very similar to the study performed by Tolu et al. on residents of physical and rehabilitation medicine in Turkey (35.4%) [3]. The low desirability of fellowship training may be due to the result of the high percentage of undecided residents (43.1%), and the most influential factor on undecided residents was performing the obligatory compulsory service following the fellowship.

In Turkey, the two fellowship-training programs for anesthesiologists, algology and intensive care, last 2 and 3 years, respectively. The most popular subspecialty was algology in our study. Although we did not ask about the reasons of their choice, we believe that it might be due to the result of a more controllable lifestyle, less work-related stress, and the lack of emergency situations and night duties.

The ratio of males was significantly higher in the group that wanted to pursue subspecialty training compared with the other two groups (*p* = 0.026 and *p* = 0.042), similar to the previous study [5]. There were no questions in our survey rendering the possible reasons of this phenomenon; it may be thought that this result was due to the greater responsibilities of women in family life (e.g. homecare, childcare, pregnancy). Also, it has been shown that women are more sensitive to burnout [6]. The compulsory service obligation factor that exists in Turkey may also keep female doctors in Turkey from undertaking additional training. During this time period, specialists may have to work in a different city away from their families, and after finishing the compulsory service period, being appointed to the city in which they want to live is not an easy situation.

Increasing postgraduate years was negatively associated with the desire to pursue fellowship, the same as in the Canadian study [2]. The presence of subspecialty divisions affected 65.5% of the respondents’ decision about pursuing subspecialty training, and it affected 52.5% of physical and rehabilitation medicine residents [3].

One of the limitations of this study is the low response rate. We were only able to reach 57.3% of the whole residents of the country (692 of 1208 residents) despite the support of related associations. The response rate of the survey was 41.04% (284 of 692 residents), which could be accepted as low considering the expected response rate of 50% in similar surveys. Due to this reality, the results of the study may not be entirely representative of all anesthesia residents in Turkey. Secondly, the existence of compulsory service in our country makes the conditions specific to our country and our results cannot be generalized to countries where compulsory service is not enforced. Thirdly, we could not compare our study with the other countries that have obligatory compulsory service programs and other specialties (e.g. military service) because we could not find any data about this in the literature.

## Conclusions

In conclusion, this study shows that the desire for sub-specialization among anesthesia residents in Turkey is unexpectedly low when compared with other countries and specialties. The result is the same as the study performed on physical and rehabilitation medicine residents in Turkey, and we think that this outcome is a consequence of the compulsory service obligation. The abolition of repeated mandatory service at each stage and the creation of new sub-specialties may increase the desire for pursuing a fellowship. We hope the preferences of anesthesia residents may help shape the health politics of the Ministry of Health and serve as a framework to document the career preferences and influencing factors of anesthesia residents in Turkey.

## Additional file

Additional file 1: The Questionnaire of Factors Affecting the Choice of Anesthesia Assistants in their Subspecialty Education and their Professional Aspects. (PDF 118 kb)

## Data Availability

The datasets used and analyzed in this study are available from the corresponding author upon request.

## Abbreviations

ARUD: Anesthesiology and Reanimation Specialists Society
SPSS: The Statistical Package for the Social Sciences
TARD: Turkish Society of Anesthesiology and Reanimation
USA: United States of America
