# Supplementary material for: Factors affecting the preference of anesthesia residents regarding subspecialty training
Source: BMC Med Educ. 2019 Sep 6;19:342. doi: 10.1186/s12909-019-1782-9 (PMC6728952; doi:10.1186/s12909-019-1782-9)
Supplement: Supplementary file 1 — The Questionnaire of Factors Affecting the Choice of Anesthesia Assistants in their Subspecialty Education and their Professional Aspects. (PDF 118 kb) [file 12909_2019_1782_MOESM1_ESM.pdf]

Factors Affecting the Choice of Anesthesia Assistants in their Subspecialty Education and their Professional Aspects

Dear Colleague,

With this survey, we aim to evaluate the preferences of your esteemed colleagues about the subspecialty education in our branch and the factors affecting them. We tried to prepare a short questionnaire that you can answer without getting bored. Thank you for your valuable contribution.

1.Sex

O Female O Male

2. How old are you

3.Your marital status?

O Married O Single

4. In what kind of hospital are you doing your residency training?

O University Hospital

O Traning and Education Hospital

O Foundation/Private University Hospital

5. Which year of residency are you in?

O 1 O 2 O 3 O 4 O 5

6. Have you participated in any scientific research during your residency except your Master's thesis?

O Yes O No

7. Have you published any articles during your residency?

O Yes O No

8. Is there an intensive care unit connected to your department/clinic in your hospital?

O Yes O No

9. If the answer to the 8. question is yes, how many beds are there in the intensive care unit?

10. Is there an algology unit connected to your department/clinic in your hospital?

O Yes O No

11. If the answer to the 10. question is yes, do you have an inpatient ward for algology patient?

O Yes O No

12 What do you think about subspecialty training?

O I would like to pursue subspecialty training in algology

O I would like to pursue subspecialty training in Intensive Care

O I don't want to pursue subspecialty training

O Not decided

13. Does the presence or absence of subspecialty units in your hospital have any effect on your decision to pursue subspecialty training?

O Yes O No

14. What is the reason if you are considering a subspecialty in Anesthesiology and Reanimation? (You can choose more than one)

O I chose it because of my personal interest

O Considering academic career

O Not considering an academic career

O Opinions of Anesthesiology and Reanimation faculty members

O Other subspecialty assistants' and anesthesia assistants' opinions about the subspecialty

O Familial and personal factors

O The city where it belongs

O Job opportunities

O Prestige

O To postpone compulsory service

O Probability of doing compulsory service in a better place

O Economic reasons (income)

O Others………………………………………….

15. What is the reason if you are not considering a subspecialty in Anesthesiology and Reanimation? (Choose only one, please)

O My personal interest in general anesthesia

O Not considering an academic career

O Opinions of Anesthesiology and Reanimation faculty members

O Other subspecialty assistants' and anesthesia assistants' opinions about the subspecialty

O Familial and personal factors

O Job opportunities

O Compulsory service at the end of the fellowship

O Economic reasons (income)

O Others………………………………………..

16. What is the reason for this if you are not sure about subspecialty?

O Absence of subspecialty unit in your hospital/clinic

O Compulsory service obligation, again

O Other……………………………………….

17. What are the factors that affect the preference of you on choosing the place for subspecialty training? (You can choose more than one)

O Having a reputation in the field

O Increased number of beds in ICU

O Inpatient ward for algology

O Advice of Anesthesiology and Reanimation faculty members

O Familial and personal factors

O To be the medical school I graduated from.

O To be the same place with my residency

O To be a place where I think I will proceed as an academician after specialization

O The city where it belongs.

O Proximity to my house

O Others……………………………….

18. After completing your specialty training, what kind of hospital do you want to work in? (After compulsory service)

O Public Hospital

O Training and Education Hospital

O University Hospital

O Foundation University Hospital

O Private Hospital

19. What are the factors that influence the place you want to work after your specialization?

O To be a place I want to live

O Familial and personal factors

O Education opportunities

O Colleagues

O Economical income

O Research opportunities

O Other……………………………………..

20. After completing your subspecialty training, what kind of hospital do you want to work in? (Only for who consider subspecialty)

O Public Hospital

O Training and Education Hospital

O University Hospital

O Foundation University Hospital

O Private Hospital/Clinic

Thank you for taking your precious time and contributing to our study.
